# Personal Health Record Software for Neuroendocrine Tumors: Patient-Centered Design Approach

**DOI:** 10.2196/68788

**Published:** 2025-06-03

**Authors:** Juan Pablo Hourcade, Michael O'Rorke, Elizabeth Chrischilles, Nicholas J Rudzianski, Brian Gryzlak, Kerry Peterman, Chris Ortman, Sam Wolstencroft, Margaret Bean, Elyse Gellerman, Josh A Mailman, Maryann Wahmann

**Affiliations:** 1 Department of Computer Science, University of Iowa, 14 MacLean Hall, Iowa City, IA, 52242, United States, 1 3193532543; 2 College of Public Health, University of Iowa, Iowa City, IA, United States; 3 Holden Comprehensive Cancer Center, University of Iowa, Iowa City, IA, United States; 4 Institute for Clinical and Translational Science, University of Iowa, Iowa City, IA, United States; 5 Healing NET, Nashville, TN, United States; 6 Neuroendocrine Tumor Research Foundation, Boston, MA, United States; 7 NorCal CarciNET Community, San Jose, CA, United States; 8 Neuroendocrine Cancer Awareness Network, Fort Mill, SC, United States

**Keywords:** personal health records, user-centered design, neuroendocrine tumors, rare disease, longitudinal research

## Abstract

**Background:**

Personal health record (PHR) software has the potential of aiding with patient engagement and data collection in longitudinal research to better understand the long-term impact of treatments on patients with rare medical conditions. Neuroendocrine tumors (NETs) represent a rare condition with unique challenges related to symptom management, treatment tracking, and patient-provider communication.

**Objective:**

This study aimed to design, develop, and evaluate PHR software tailored for patients with NETs as part of a longitudinal research study. Our goal was to create a patient-centered PHR that supports both self-management and research data collection.

**Methods:**

This included activities spanning the entire development lifecycle from identifying user requirements through focus groups and surveys, iterative prototype refinement via cognitive walkthroughs, and usability testing of the functional PHR system. Feedback from patient advocacy organizations and clinical experts further informed PHR development.

**Results:**

The resulting PHR allows patients with NETs to access condition-specific information, track symptoms, monitor treatment regimens, and share data with health care providers. Patients valued the ability to visualize personal health trends and patterns over time, enhancing both self-management and communication with medical teams. Usability testing indicated high levels of patient satisfaction with the system’s functionality and design.

**Conclusions:**

The development of this PHR demonstrates the value of engaging patients in the design process to ensure that health technologies address real-world needs. Our approach provides a model for designing PHR systems for other rare conditions, highlighting the importance of patient-centered design in supporting both clinical care and longitudinal research.

## Introduction

The past 20 years have seen a revolution in the digitization of health records [1-3]. There are 2 aspects to this digitization that have been occurring in parallel. One is the advent of electronic health records in hospitals and doctor’s offices [1,3], which are often accompanied by health portals providing patients with access to their health records [4,5]. The data in these systems almost exclusively originate from hospitals, laboratories, and doctor’s offices. The other is patients using technology to capture or record personal health data. This latter aspect has involved automated capture of information (eg, steps taken from smartwatches or smartphones) [6], the use of smart health devices (eg, blood pressure monitors that connect with smartphones or computers) [7], and the use of personal health record (PHR) software that enables patients to record information that may otherwise not be tracked (eg, symptoms) [8,9].

PHR software has also been proposed as a tool to facilitate medical research [10]. Many medical studies require long-term follow-up of patients with self-reported measures. The data collection necessary for such studies has traditionally involved mailing paper questionnaires to patients using the postal system or calling them on the phone. Using PHR software for the same purpose has the potential of lowering costs by avoiding mailings, transcriptions, or time spent on phone calls, while increasing data quality by avoiding errors in transcription and enabling patients to contribute data at a time and location and on a device that is most convenient for them [11,12]. In order for PHR software to successfully play a data gathering role for research studies it needs to provide a valuable service to patients, otherwise patients are less likely to use it [9,13]. Ideally, PHR software used for research studies would follow a model similar to that followed by free online services: users get a valuable service in exchange for their personal data. The difference in this case is that patients would explicitly consent to provide their data for a specific purpose ie, medical research.

One specific area of medical research that we believe could benefit from the use of PHR software for gathering data from patients is research on rare medical conditions. Knowledge gaps remain prevalent for most rare medical conditions due to systemic and structural challenges in health care and research. These include small, geographically dispersed patient populations, limited funding for research, and frequent diagnostic delays. The latter arise from the complexity of these diseases, limited practitioner training when compared to more common conditions [14], and the evolving understanding of disease characteristics [15]. There is also often a need for longitudinal research to better understand the long-term impact of treatments on patients with rare medical conditions [16] and PHR software could play a role in repeated data collection for such research. The challenge is that because these conditions are rare, it is unlikely that there are commercial PHR systems that are a good match for patient needs, unlike the case for more common conditions, such as asthma, diabetes, glaucoma, HIV, hyperlipidemia, and hypertension [14,17]. The sole example we found of a PHR designed for patients with a rare condition (idiopathic thrombocytopenic purpura) resulted from an adaptation of a generic PHR system based on advice from expert physicians [18]. We recommend, in addition to consulting with experts, engaging with patients in user-centered design activities, positioning patients as research partners.

In this article, we present our experience engaging with patients with neuroendocrine tumors (NETs) [19,20], a rare medical condition, throughout the design process of PHR software currently being used as part of a prospective cohort study of people with NETs [19-21]. NETs include a broad variety of tumors that can appear throughout the body, but are most common in the small intestine, pancreas, and lung [19,20].

## Methods

### Participant Recruitment

For all phases of the project, we recruited patients with NETs through 4 partnering NET advocacy groups in the United States (The Neuroendocrine Tumor Research Foundation, The Neuroendocrine Cancer Awareness Network, The Healing NET Foundation and Northern California CarciNET Community). Inclusion and exclusion criteria for volunteers in this study included adults (≥18 years old, any gender) with a confirmed diagnosis of any NET, able to read, speak, and understand English to participate in focus groups, surveys, and usability testing, a willingness to share personal experiences regarding symptom tracking, treatment management, and interactions with health care providers related to their NET, and access to a computer, tablet, or smartphone with internet connectivity to interact with the PHR system and participate in online activities. There were no other demographic or other requirements for volunteer participants including previous PHR experience. Overall, 89 patients with NETs participated in at least 1 activity (60 female, 18 male, 11 unknown gender; 75 White or Caucasian, 1 Black or African American, 1 Asian or Asian American, 12 unknown race; 2 Hispanic or Latino, 80 not Hispanic or Latino, 7 unknown; 32 with postgraduate degrees, 27 with college degrees, 16 with some college, 2 with a high school degree or GED, 1 without a high school degree or GED, 11 unknown educational level; 7 aged 18‐40, 30 aged 41‐60, 49 aged 61‐80, and 3 aged >80 years). Participants who provided their home address (n=75) joined activities from 34 different states and one location outside of the United States.

### Outline of Research Activities

Research activities involved a comprehensive, multiphase process designed to engage patients with NETs at every stage of the PHR development including: (1) 9 online design sessions with patients with NETs to develop requirements and a survey to validate these requirements with a broader set of patients, (2) another 9 online sessions to iteratively improve a low-fidelity interactive prototype, and (3) three sets of online usability testing sessions to refine the implemented PHR software (refer to Table 1 for details). The patient activities described in this report were reviewed and approved by the University of Iowa Institutional Review Board.

**Table 1.** Session activities organized by phase, strategy, format, and number of participants.

Strategy		Format	Participants, n	Activities
Phase 1: identifying requirements				
	Brainstorming	Online focus groups (3 groups of people, first of 3 sessions)	16	Imagine a superhero that can help with their condition – what would the superhero do? (Superhero construct frees patients from perceived constraints) Research team compiles a list of potential features from participant discussions
	Organizing	Online focus groups (same 3 groups of people, second session)	16	Participants rank desirable or undesirable features Prioritize features and provide more details on what they want Research team reviews results and identifies what categories to implement in the NET^a^ PHR^b^ in two areas: (1) providing information on NETs; (2) tracking condition-related data
	Organizing	Survey of focus group attendees	16	Further prioritization of features and details on requirements
	Confirmation	Online focus groups (same 3 groups of people, third session)	16	Discuss survey results with each group to better understand the reasons behind survey answers Probe what patients would expect from a PHR that implemented these requirements
	Ranking and attitudes	Validation survey	52	Prioritize categories of information or advice (high, intermediate, and low) Preference of categories to track
Phase 2: prototype refinement				
	Rapid prototyping	Cognitive walkthrough with users	27 (3 groups of 9)	Rapid prototyping with wireframe software (Balsamiq) by research team Patients provide feedback on prototype based on specific tasks Iteratively improve between group activities until optimal design reached (3 rounds with each group of 9 patients)
Phase 3: usability testing				
	Functional prototype	Usability testing (tasks, survey)	10	Use functional prototype on Zoom with team Patients complete prepared tasks using a test version of the tool, thinking aloud Provide feedback via survey—NASA task load index administered after each task Discussion at end on what worked well, what did not, and what they would change Iteratively improve based on feedback (3 rounds of usability testing)

a NET: neuroendocrine tumors.

b PHR: personal health record.

### Ethical Considerations

We obtained ethics approval from the University of Iowa’s Institutional Review Board (202110283). All participants received an information and consent letter before enrollment and participation. Participants were compensated US $50 for Phase 1 and Phase 2 Zoom sessions, US $10 for the Phase 1 survey, and US $20 for Phase 3 usability Zoom sessions. To preserve participants’ privacy and confidentiality, we took notes rather than record sessions and these notes are not associated with specific participants. In addition, the Phase 1 surveys were designed to ensure anonymity of participants by not collecting personally identifiable information.

### Phase 1: Identifying Requirements (Online Focus Groups, 9 Sessions)

To identify requirements, we recruited 16 patients with NETs, divided them into 3 groups, and conducted 3 1-hour online sessions with each group using Zoom, for a total of 9 sessions.

For the first session, our goal was to obtain ideas for PHR software for patients with NETs without any constraints. After introducing the project and our overall goal, our instructions to patients during the first session were to imagine that there was a superhero that could help them with their medical condition, who could do anything except give them a cure. The idea behind the superhero discussion was to remove any constraints patients could think of with respect to what would be possible with technologies. When we met with subsequent groups, we started by getting ideas from them and then introducing ideas by previous groups if they had not mentioned them to obtain further feedback. We had previously used the superhero activity, albeit face-to-face, while developing a PHR focused on elder medication safety [13] and others later used it face-to-face with older adults as well [22].

During each superhero session, at least 3 researchers took notes on participants’ ideas. To analyze these notes, we used an inductive coding process, similar to the one proposed by Gioia et al [23]. First, 2 of the researchers (JPH and KP) went through the notes and created 1 physical sticky note per unique concept, for a total of 51. Using these sticky notes, they then organized patient ideas, developing an affinity diagram, from which they produced a hierarchically organized list of requirements. The high-level themes in this hierarchy of requirements were information on NETs (eg, support groups, advocacy, education, and ideas for relief), tracking condition-related personal information, and managing logistics. These requirements were the basis for the second session with each group. We used this second session to validate the set of requirements, begin to prioritize them, and obtain more details on the requirements. We had pursued a similar approach in previous research, in particular with respect to information tracking, to understand the difference between information patients would like to track and information they would be willing to enter [13].

The research team met after the second set of sessions to consider the priorities identified by patients and the practical realities of what we could accomplish within the constraints of the project. One important area in which patients were interested that we were unable to pursue was managing logistics, from better integration of electronic health records across providers to managing prescriptions and appointments. Based on these considerations, we identified a set of requirements on which to focus. We then developed a web-based Qualtrics survey sent to the 16 participating patients with NETs to obtain more details on requirements and further prioritize them. We used the third set of sessions to discuss the results of the survey with each of the groups to better understand the reasons behind survey answers and get a sense for what patients would expect from PHR software that implemented these requirements.

After the third set of sessions, we created an electronic survey to be distributed to a broader set of patients with NETs to validate the requirements we developed with 16 patients. We believe it is important to validate requirements from participatory sessions with a larger group of patients due to potential biases related to who can be available and feel comfortable participating in such activities. Others have followed similar approaches to balance the depth of discussion in participatory sessions with the greater representativeness afforded by a survey [24]. Questions focused on preferred data tracking features, information needs, and feedback or output options (eg, personal trends and data visualizations). The 52 patients with NETs who answered the survey helped us validate and further prioritize the set of requirements we had developed. The result of Phase 1 was a list of specifications that would guide prototype development.

### Phase 2: Prototype Refinement (Cognitive Walkthroughs, 3 Rounds, and 27 Participants)

Our next step was to design an interactive wireframe prototype using Balsamiq [25]. Balsamiq enables screen designs that look as if they were sketched with pen and paper and includes commonly used user interface elements (eg, buttons and images). The sketches are created so feedback on them can focus on those elements and navigation through the system, as opposed to aesthetic aspects. Screen sketches can be linked to each other, so the prototype is navigated by clicking on user interface elements (eg, menus and links) like a normal website.

We then sought to iteratively improve the prototype through 3 rounds of feedback from patients with NETs over Zoom using cognitive walkthrough with users techniques [26], which have previously been applied to wireframe prototypes [27]. We obtained feedback from 3 different groups of 9 patients with NETs, for a total of 27 subjects. Patient participation varied per round. For the first round, we had 24 patients participate, 20 for the second, and 17 for the third. During each session, a research team member would work with 2‐3 patients at a time (ie, small group sessions), asking them how they would navigate the system for a given set of tasks. Participants verbalized their thought process (“think-aloud” protocol), highlighting usability issues and areas of confusion. Feedback from each round informed changes to the prototype (ie, iterative refinement), which were then reevaluated in subsequent rounds.

### Phase 3: Usability Testing (3 Rounds, 10 Participants)

The following step was to develop a working web application based on the design of the Balsamiq prototype. This design incorporated more detailed functionality that could not be tested in Balsamiq and aesthetic aspects, such as fonts and colors. To implement the design, we used an existing system we previously developed, the Iowa PHR, and customized it based on the outcomes of Phases 1 and 2. The Iowa PHR is a Java 8 [28] Spring application [29] with a Microsoft SQL Server database [30]. It is hosted with Jetty [31] and fronted with Apache [32].

To iteratively improve this working system, we conducted 3 rounds of usability testing [33] with a total of 10 patients with NETs, 4 participated in the first session, 3 in the second, and 3 in the third. There were about 3 weeks between the first and second round of sessions, and about 6 months between the second and third rounds (in part due to competing commitments for the software developers), enabling our developers to make changes to the system.

At each session we asked participants to complete tasks involving the system’s most important features, including finding specific types of information about NETs and tracking a variety of data. After a group of tasks pertaining to similar features, we asked patients to fill out a NASA Task Load Index survey [34] to assess the ease or difficulty of those tasks. At the end of each session, patients provided qualitative feedback on what worked well, what did not work well, and what they would like to change about the system. From this feedback, after each round of usability sessions, we developed a list of changes for the PHR, which the development team completed before the following round of usability sessions.

Our patient advocacy organizations provided “test-drive” feedback through credentialed access to the PHR, followed by structured surveys (via Research Electronic Data Capture [REDCap]) [35] to capture their insights. In a similar way, useful feedback was also obtained from our NET specialists (ie, working oncologists and endocrinologists) who reviewed the PHR to suggest improvements related to medical terminology, treatments, information accuracy, and ordering or presentation of data.

After we incorporated all feedback into the PHR, we made it available to patients with NETs enrolled in an ongoing research study in March 2024. Subjects enrolled at the time were sent an email notification introducing the availability of those features. Further outreach is planned to promote awareness and gather more comprehensive feedback on long-term utility.

## Results

### Phase 1: Identifying Requirements

#### Identified Requirements

The requirements we identified fell into 2 categories: information on NETs and tracking condition-related personal information. The types of information in which patients were interested were related to the fact that NETs are rare, there are different subtypes of NETs, and research is still ongoing regarding optimum therapeutic management and treatment sequencing [19,20].

In terms of tracking personal information, most patients had a strong interest in tracking symptoms because some (eg, diarrhea) have a significant impact on quality of life [20]. Together with symptoms, they expressed interest in tracking factors possibly triggering symptoms including consumption of certain foods, medications, episodes of stress, and quality of sleep. They sought to share this information with their medical team but also visualize patterns and potential interactions over time.

#### Validated Requirements

In total, 52 patients with NETs completed the requirements validation survey. The survey focused on the 2 key aspects that emerged from our participatory sessions: providing information on NETs and tracking condition-related data. Through the survey, we were able to both validate and prioritize the requirements obtained during Zoom sessions. Multimedia Appendix 1 shows preferred types of data to track and Table 2 lists preferred features related to tracking data. Multimedia Appendix 2 shows preferred types of information, education, or advice about NETs.

**Table 2.** How useful are certain feedback and output options for patients with neuroendocrine tumors.

Feedback or output option	Most useful, n	Least useful, n
Personal trends over time	39	1
Patterns	29	3
Comparisons of data from other users	13	19
Ability for other users to enter data	14	11
Simple graphs or charts	26	5
Two or multi axis graphs or charts between items	11	14
Printable data, graphs, or charts	25	6
Download raw data	11	21
Detailed reports and summaries	25	6
Allow users to select type of output	26	3
Snapshots or focused summaries	17	8
Option to save editable preferences	15	12
Flexibility in timeframe of outputs	12	13
Generic notifications or nudges	13	18

### Phase 2: Prototype Refinement

The most difficult challenges we addressed through these sessions involved information architecture for the broad range of information patients thought should be provided about NETs. Due to the wealth of information available, challenges included decisions on the most reliable or updated data sources, and how to organize and categorize the information. In addition, we also obtained feedback on basic tracking capabilities during the first prototyping round, and on custom tracking features (enabling users to add their own custom tracking categories) during the second and third rounds.

Iteratively improving these interactive wireframe prototypes in Balsamiq enabled us to improve the design of the system at a low cost, since it is quick and easy to modify Balsamiq prototypes when compared to modifying a web application.

### Phase 3: Usability Testing

Patients were provided with a set of 10 predetermined tasks, presented in an order of increasing complexity, concerning information seeking, data entry, and editing and deleting entries. At the end of each set of related tasks, patients were instructed to complete the NASA Task Load Index survey [34] which evaluated patients’ responses across 6 dimensions including mental, physical and temporal demand as well as ease of performance, effort involved and frustration with the set tasks. Overall, the findings indicated a generally manageable task load, with the majority of patients scoring mental, physical and temporal demands across tasks negatively (ie, low demand). As expected, higher order tasks were associated with an increase in demands of effort, but most patients reported minimal frustration with the assigned tasks.

Together with the feedback obtained from our patient advocacy organization partners and NET clinician groups, usability testing feedback helped us identify minor bugs, make some information easier to find, make it easier for users to start tracking data, simplify some tracking options, and otherwise confirm that the system was ready for use.

The final version of the PHR app includes options for tracking data related to the condition. We identified 18 categories of data from our interactions with stakeholders (eg, skin flushing, food intake, pain, and medication intake), which are ready to be used with a design that aims to minimize the steps required to enter data. In addition, users can create their own custom category to track, which addresses the broad range of needs within NETs. An instructional video is available to patients explaining the PHR app’s tracking capabilities and how to use them. The system also enables users to track the medications they are currently taking or have previously taken, with a user interface developed during previous research [10,13]. In addition, the PHR app also provides access to various types of information relevant to patients with NETs organized into tabs: about NETs, treatments for NETs, tips for managing care (many of these came from stakeholders and patients), and NET resources (eg, patient support groups and clinical trials).

A video walkthrough of the final PHR is available in the Multimedia Appendix 3.

### Utility Assessment

Our PHR can now be accessed by patients with NETs enrolled in a research study where the emphasis is on completing study-related surveys. Patients enrolled in the study received one email announcing the newly designed features of the PHR. These 1070 patients had access to the PHR, mostly as they waited completion of a subsequent study survey, with 110 logging in just to use the PHR. These 110 patients were the most active in using it. We are planning to further advertise the PHR to promote greater awareness for eligible patients.

## Discussion

### Principal Findings

As a result of extensive focus group-based participatory design and usability testing, we learned that patients with NETs were interested in keeping track of their symptoms and accessing many types of information about their disease, as well as the means to identify personal patterns, interactions and trends in these factors over time. Having the capacity to share these observations more efficiently with their health care providers was of high importance to them. They frequently expressed that they had to become advocates for their own disease management and that these features would help them communicate better with their providers. These findings were different from our previous study designing a PHR with older adults [10,13]. Participants often voiced their appreciation for being treated as partners in the design process, their feedback helped identify navigation issues, data visualization preferences, and feature requests. Their input and improvements based on real-time feedback were crucial to optimizing the PHR.

Rare diseases present a unique set of challenges for patients, health care providers, researchers, and society [36,37]. Rare diseases are often difficult to diagnose because many medical professionals are unfamiliar with them. Patients frequently go through a “diagnostic odyssey” that can take years, seeing multiple specialists before receiving an accurate diagnosis [38]. Moreover, symptoms of rare diseases often overlap with more common conditions, leading to misdiagnosis and inappropriate treatment. Since rare diseases affect a small percentage of the population, there is often less financial incentive for pharmaceutical companies to develop treatments, a phenomenon known as the “orphan drug” problem [39]. Many rare diseases have no approved treatments or cures, leaving patients with limited options for management and quality of life improvement. Specialists for rare diseases are often concentrated in a few medical centers worldwide (ie, centers of excellence), meaning patients may need to travel extensively to receive appropriate care or consultation. In particular, rural areas may lack access to these experts, further isolating patients and delaying necessary care. Furthermore, the rarity of these diseases can mean less public and governmental awareness, leading to fewer resources and funding opportunities for research, support services, and patient care. This has promoted the evolution and development of many patient advocacy organizations, often championed and spearheaded by patients with rare disease and their families in response to their own patient experiences. These groups are essential for raising awareness, driving research and elevating health-care standards, but often have limited resources themselves [40]. Unfortunately, this forces many patients with rare disease to advocate for their own disease, and their particular needs, whether they be informational, expertise or treatment related, physical or emotional. Due to the small patient populations, and often limited data available on them, protecting patient privacy while sharing valuable information for research also presents unique challenges in rare disease studies, and highlights the importance and potential of a coordinated effort between health care organizations, researchers, patient advocacy groups and industry to address these challenges.

NETs in particular are an excellent case-study among rare conditions for the development of a PHR. They are typically slow growing in nature with an unusually prolonged survival and significant symptom burden [41]. Their vague antecedent signs and symptoms cause significant delay and difficulty in their diagnosis and detection [19]. Metastatic disease is observed in up to 40% of NETs at initial diagnosis [42], and many patients with liver or other distant metastases experience carcinoid syndrome, a direct result of serotonin hypersecretion [43] manifesting clinical symptoms ranging from watery diarrhea and flushing to bronchospasm, hypotension and right-sided cardiac deficits (carcinoid heart disease). Thus, patients with NETs typically experience prolonged survival with active disease, and many have significant symptom burdens that may vary daily [44]. Treatment strategies for NETs depend on tumor grade, stage, location, and functional status. Usual treatment for low to intermediate grade gastroenteropancreatic (GEP-NETs, the most common NET subtype) remains surgery, but the requirement for adjuvant therapy is questionable [45-47]. Therapy with somatostatin analogues are established first-line agents for low-grade GEP-NETs, with demonstrated improvements in overall survival [48,49]. However, there are no clear consensus guidelines as to the optimum sequencing of other therapeutic options [50-52], leading many patients to read widely, and become advocates for their own disease and its appropriate management.

To our knowledge, only one previous study has been published evaluating the expansion of a web-based PHR in another rare disease setting. This was a 6-month prospective pilot study involving 43 patients diagnosed with chronic idiopathic thrombocytopenic purpura (ITP) in Marseille, France [18]. The primary objective of the study was to evaluate the usability of the Sanoia tool (a freely accessible web-based PHR) that was expanded with ITP-specific tools including links to validated ITP-related web resources, an emergency protocol based on updated national recommendations, and a section for personal notes, allowing patients to record symptoms, medications, and other relevant events. Health evaluations undertaken and assessed with the Sanoia Interface included quality of life (via an ITP Patient Assessment Questionnaire) and usability of the tool (measured by the frequency of tool usage and patient engagement with the personal notes section). The pilot study demonstrated good usability of the customized Sanoia interface for patients with ITP. While no significant impact on quality of life was observed, the tool facilitated patient engagement and information sharing with health care providers [18].

In comparison, our adaptation of the Iowa PHR was more extensive – whilst we similarly linked out to resources for patients with NETs, we spent more effort in development of new user interfaces to physically capture, visualize and document disease symptoms, track and compare these features over time, and record past and present medication use. An advantage of the Sanoia interface is that patients could create their PHR, receive a unique personal identifier, and share this identifier with health care providers to facilitate access to their medical information in multiple languages.

When compared to generic PHRs, ours shares functionalities including medication management, education, and self-health monitoring [53]. Unlike some generic PHRs, it is not integrated with a hospital’s or health system’s records and therefore does not enable patients to access their health records, communicate directly with health care providers, or make appointments [53]. The patients with whom we interacted, all based in the United States, typically had to interact with multiple providers who often used different electronic health records systems. Hence, a PHR that could integrate with all these systems would require significant resources to implement and maintain, making these features beyond the scope of our project. Our PHR, however, addresses an aspect that is missing from most PHRs: personalization [53]. Our PHR is personalized not only to the specific needs of patients with NETs but also gives them the opportunity to further customize what information to track. Addressing patients with NETs needs not only makes the PHR more relevant to patients with NETs, but also more usable by reducing the number of steps necessary to access relevant information and functionality, when compared to a generic PHR.

Anticipated long-term impacts of the PHR could include future updates to extend the current tracking capabilities including refining symptom tracking, medication logs, and data visualization tools, making them more customizable and user-friendly. Adaptive user interfaces may also be possible, offering personalized dashboards and automated data entry suggestions. Similar to the Sanoia interface, a major future development would be integrating with hospital and clinic-based EHRs, allowing seamless data sharing between patients and providers. The PHR’s ability to collect patient-reported data over time could contribute significantly to other research studies on rare diseases (ie, future cross-disease adaptations). Establishing ongoing engagement through user feedback surveys, focus groups, and iterative testing to refine features in response to user experiences would close the feedback loop and augment on-going user support and engagement. It may also be beneficial to consider further data privacy and security enhancements (ie, strengthening encryption, access control, and data-sharing policies to ensure continued regulatory compliance).

Overall, developing PHR software tailored to rare disease patients can offer numerous benefits, improving health care experiences, treatment outcomes, and providing research opportunities. Patients with rare disease and their caregivers often play an active role in managing their care. A PHR could provide them direct access to their medical data making it easier to monitor changes, track symptoms, and communicate with health care providers. Importantly, a PHR has the potential to consolidate all relevant health data—such as diagnoses, test results, medications, and treatment plans—into a single, easily accessible platform (ie, centralized medical information). This empowers patients with access to their own health information, promotes self-advocacy and informed decision-making.

### Strengths

Through patient-centered design approaches, this project prioritized patient involvement throughout, ensuring that the final PHR addressed real patient needs effectively. Multiple phases of testing and refinement (focus groups, surveys, and usability testing) lead to a well-validated and user-friendly PHR system adapted for a NET population. The final PHR includes symptom tracking, custom tracking options, and access to relevant information and resources for patients with NETs, addressing both immediate and long-term patient information needs. Use of Balsamiq for wireframe development allowed for efficient and cost-effective testing of ideas before full implementation. Our emphasis on NETs addresses a critical gap in health care for rare medical conditions, which are often underserved. As a result, patients in the study have gained a tool that improves their understanding and management of their condition, empowering self-advocacy. The final adapted PHR facilitates longitudinal data collection, benefiting both medical research and patient care, a platform that could easily be tailored to other rare disease applications in similar research settings.

### Limitations

A limitation is that the study PHR does not have interoperability with patient medical records or smart health devices. Features that integrate patient reported information with systems-derived clinical records should be developed. Incidentally, patients who participated in Phase 1 often had multiple providers and medical record systems. They reported significant challenges with lack of interoperability across their medical records from different providers.

NETs are not necessarily representative of other rare medical conditions. Simpler or more complex patient needs could result in a need to adjust the methods we presented. Despite the features, only 110 out of 1070 patients logged in specifically to use the PHR, suggesting a need for greater awareness and engagement efforts which we plan to promote. Although valuable, the insights may be skewed by the demographics and availability of the participants involved, potentially overlooking the perspectives of less active or digitally less literate patients.

### Conclusions

PHR software can be a useful tool to collect data from patients with their permission as part of research studies while providing them with a valuable service. This strategy could be particularly useful for studies of rare medical conditions, where commercial PHR software may not be available and there is often a need to identify the most effective treatments and optimal therapy sequencing. We presented our experience designing and developing PHR software designed for patients with NETs, a rare medical condition. Our online, patient-centered approach altogether involved 89 patients with NETs in identifying requirements through participatory activities, validating them through a survey, providing feedback on an interactive wireframe prototype, and usability testing a fully implemented system. Not only was patient input valuable in helping us design a useful, usable system, but many of the participating patients enjoyed the activities, having their opinions valued, and in many cases meeting other patients with NETs for the first time. We expect that the methods we used would work well for similar projects.

## Supplementary material

10.2196/68788 Multimedia Appendix 1 Ranking of interest in tracking data (first, second, and third preferences) by patients with neuroendocrine tumor during validation of requirements.

10.2196/68788 Multimedia Appendix 2 Ranking of the types of information and education and advice that could be provided to newly diagnosed patients.

10.2196/68788 Multimedia Appendix 3 NETPRO guide.

## Abbreviations

GEP-NET: gastroenteropancreatic neuroendocrine tumor
ITP: idiopathic thrombocytopenic purpura
NET: neuroendocrine tumor
PHR: personal health record
REDCap: Research Electronic Data Capture
