# Supplementary figures and images for: Personal Health Record Software for Neuroendocrine Tumors: Patient-Centered Design Approach
Source: JMIR Hum Factors. 2025 Jun 3;12:e68788. doi: 10.2196/68788 (PMC12151452; doi:10.2196/68788)

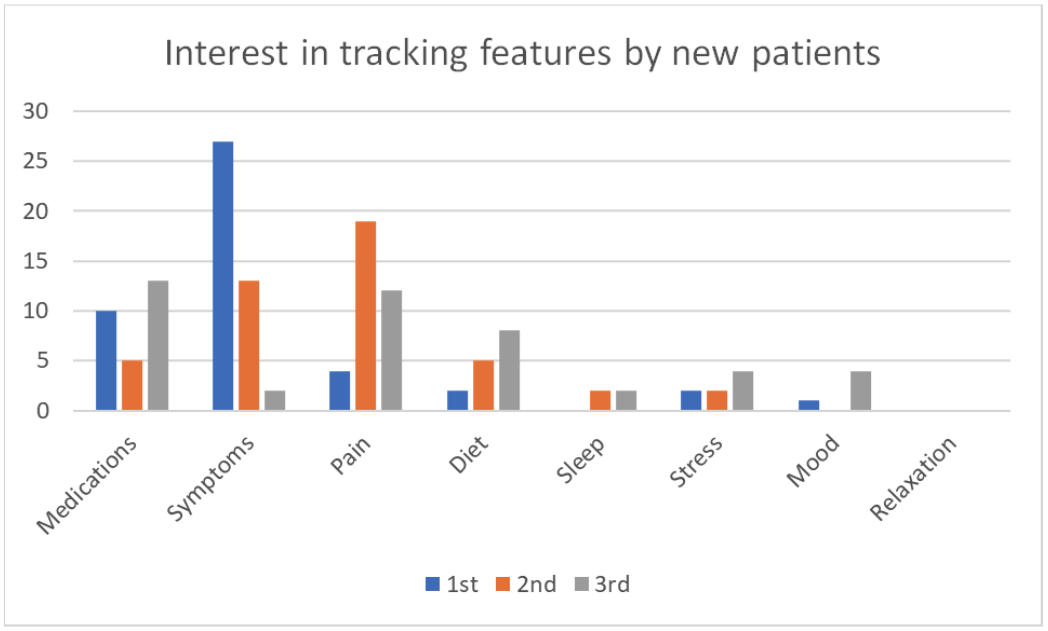

Supplement: Multimedia Appendix 1 [file humanfactors-v12-e68788-s001.png]

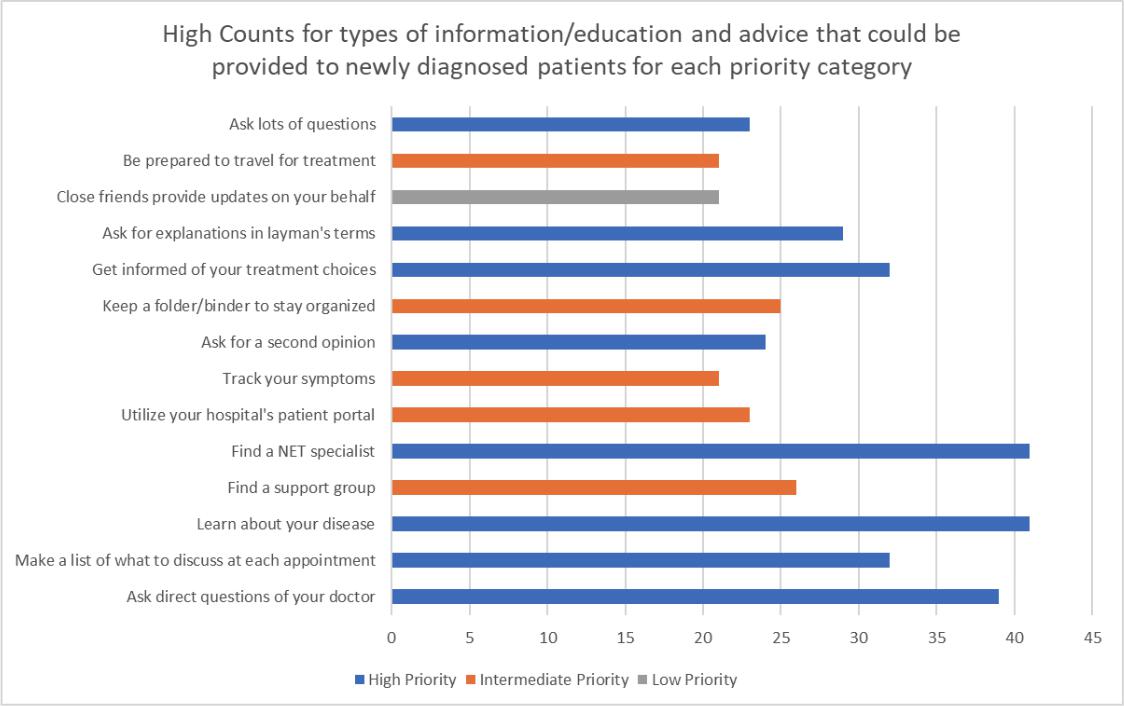

Supplement: Multimedia Appendix 2 [file humanfactors-v12-e68788-s002.jpeg]
